# Factors influencing the length of stay among patients admitted to a tertiary pediatric intensive care unit in Saudi Arabia

**DOI:** 10.3389/fped.2022.1093160

**Published:** 2022-12-19

**Authors:** Reem Alshaikh, Ahmed AlKhalifah, Amel Fayed, Sawsan AlYousef

**Affiliations:** ^1^ General Pediatric Department, King Abdullah bin Abdulaziz University Hospital, Riyadh, Saudi Arabia; ^2^ Pediatric Intensive Care Unit, Qatif Central Hospital, Qatif, Saudi Arabia; ^3^ Clinical Sciences Department, Princess Nourah Bint Abdulrahman University, Riyadh, Saudi Arabia; ^4^ Pediatric Intensive Care Unit, Children's Specialized Hospital, King Fahad Medical City, Riyadh, Saudi Arabia

**Keywords:** length of stay, intensive care units, pediatric, multivariate analysis, complications, infection

## Abstract

This study aimed to assess the variables contributing to the length of stay in the pediatric intensive care unit. This study utilized a retrospective design by analyzing data from the Virtual Pediatric Systems web-based database. The study was conducted in a tertiary hospital—King Fahad Medical City in Riyadh, Saudi Arabia—from January 1, 2014 to December 31, 2019. The patients were admitted to intensive care with complex medical and surgical diseases. The variables were divided into quantitative and qualitative parameters, including patient data, Pediatric Risk of Mortality III score, and complications. Data from 3,396 admissions were analyzed. In this cohort, the median and mean length of stay were 2.8 (interquartile range, 1.08–7.04) and 7.43 (standard deviation, 14.34) days, respectively. The majority of long-stay patients—defined as those staying longer than 30 days—were less than 12 months of age (44.79%), had lower growth parameters (*p* < 0.001), and had a history of admission to pediatric intensive care units. Moreover, the majority of long-stay patients primarily suffered from respiratory diseases (51.53%) and had comorbidities and complications during their stay (*p* < 0.001). Multivariate analysis of all variables revealed that central line-associated bloodstream infections (*p* < 0.001), external ventricular drain insertion (*p* < 0.005), tracheostomy (*p* < 0.001), and use of mechanical ventilation (*p* < 0.001) had the most significant associations with a longer stay in the pediatric intensive care unit. The factors associated with longer stays included the admission source, central nervous system disease comorbidity, and procedures performed during the stay. Factors such as respiratory support were also associated with prolonged intensive care unit stays.

## Introduction

Pediatric intensive care unit (PICU) admissions account for a substantial number of admissions every year among the pediatric population. The average length of stay (LOS) for PICU patients differs across demographic regions and healthcare centers. This difference is denoted by the variability in diseases, population characteristics, healthcare services, and intensivists' expertise (1, 2). The average LOS for a PICU patient is greater than seven days in 40% of admitted patients (3).

This study examined multiple variables contributing to the LOS. Previous studies identified several factors that contribute to the LOS including patient age, severity of illness, presence of nosocomial infections, acute kidney injury, cardiorespiratory disease, post-cardiac intervention patients, and patients with cardiac arrest before admission (4–13). Complications related to clinical conditions, such as drug-drug interactions and hospital-acquired infections (HAIs), are also associated with prolonged LOS (10, 14).

Significant effort has been made to decrease LOS and associated costs (14–17). An increase in availability of bedside nurse practitioners as well as the implementation of sedation-tapering protocols and other projects have resulted in a significant decline in LOS (17, 18). Discrepancies in demographic data, type of disease, hospital care protocols, intervention, and experienced intensivists' availability between PICUs are some factors that have been previously used in attempts to form an accurate predictive model for LOS for all PICUs worldwide (1, 13, 19). From Saudi Arabia, in particular, there are limited data regarding the factors influencing LOS. Accordingly, this study describes the factors influencing LOS in a tertiary PICU in King Fahad Medical City (KFMC). Identifying these factors will help develop targeted research aimed at reducing the length of stay in the PICU. Such studies from different geographic areas involving different populations and clinical practices will add to the variety of data in the literature, which will make future meta-analyses and predictive models more generalizable.

## Materials and methods

### Study design and settings

This retrospective study used the Virtual Pediatric Systems (VPS) web-based database (Los Angeles, CA; http://www.myvps.org). The data were entered into the VPS by a trained medical records officer and reviewed after retrieval by researchers (pediatric intensive care physicians). Institutional review board (IRB) approval was obtained from the local IRB committee at King Fahad Medical City (IRB log number: 21-355).

The KFMC is a tertiary hospital with a multidisciplinary PICU that provides specialized care and serves as a referral center for other regional healthcare institutes. The patients were admitted to the KFMC PICU with complex medical and surgical diseases.

All children younger than 14 years of age admitted to the KFMC PICU between January 1, 2014, and December 31, 2019, were included in this study. Patients older than 14 years and neonates with a corrected gestational age of <44 weeks were admitted to different units and were not included in the VPS database. Patients with incomplete data and those still admitted to the PICU at the end of the study period were excluded from the study because LOS could not be determined.

The data collected included baseline characteristics, LOS in the PICU, patient origin, history of previous PICU admission, diagnosis, comorbidities at admission, and Pediatric Risk of Mortality (PRISM) III, which calculated the probability of death. PRISM III is a score developed in 1996 and is calculated based on 21 physiological variables to estimate the severity of illness and probability of death (2). Other admission-related variables were collected, including procedures such as continuous renal replacement therapy; central venous catheter (CVC) insertion; exchange transfusion; Foley catheter insertion; intracranial pressure (ICP) monitor/extra-ventricular drain (EVD) insertion; peripherally inserted central venous catheter (PICC); tracheostomy and arterial line insertion; respiratory support needed; cardiac arrest events and complications such as ventilator-associated pneumonia (VAP), central line-associated bloodstream infection (CLABSI), catheter-associated urinary tract infection (CAUTI), and hospital-acquired pneumonia (HAP) acquired during admission. Information on do not resuscitate (DNR) and mortality status was also collected.

### Defining the length of stay

The LOS was calculated by subtracting the date and time of PICU admission from the date and time of PICU medical discharge. The PICU physical discharge time was excluded to avoid the influence of logistic causes that were not documented as a reason for the delay in patient charts. As previous studies have concluded, prolonged LOS was defined based on the 95th percentile for patients included in this study (6). The 95th percentile was calculated to be 29.5 days in this study population.

### Statistical analysis

After analyzing the distribution of LOS, the patients were stratified into normal and prolonged stay groups. The baseline characteristics were compared between the two groups. The median and interquartile range (IQR) were calculated for continuous numerical data, whereas frequencies and percentages were used for nominal data. Mann-Whitney U, Kruskal-Wallis, and Chi-square tests were used to compare the characteristics of patients who stayed for 30 days or less and those with prolonged LOS. Statistical *significance was set at P < 0.05*.

Multivariate analysis was performed using logistic regression analysis. Variables were found to be significantly different (*p* < 0.05) between the two groups included in the multivariate analysis. The odds ratio (OR) was estimated with a 95% confidence interval (CI) for each included variable presumed to contribute to increasing length of stay.

Data were collected and analyzed using IBM SPSS Statistics (version 25.0; IBM Corp., Armonk, NY, USA). The statistical analyses performed in this study were thoroughly reviewed by a trained biostatistician.

## Results

In this study, there were 3,969 PICU admissions during the study period. Of these admissions, 573 (14.4%) were excluded because of missing data. Thus, 3,396 admissions were recorded and analyzed.

### Length of stay definition

LOS was found to have a rightward skewed distribution with a median of 2.8 (IQR, 1.08–7.04) and a mean of 7.43 ± 14.34 days. The 95th percentile was calculated to be 29.5 days. Thus, LSPs were defined as patients who stayed in the PICU for > 30 days.

### Patients and admission characteristics

The duration of admission was measured at 0–30 days for 3,233 (95.2%) patients and > 30 days for 163 (4.8%) patients. 44.8% of LSPs were less than 12 months of age (Table 1). LSPs had significantly lower growth parameters than patients with shorter stays (height of 73 cm and 87 cm, respectively, and weight of 8 kgs and 11.2 kgs, respectively) with a *p*-value of < 0.001. The sex of the admitted patients did not differ significantly between the groups (See Table 1 below).

**Table 1:** Patient characteristics in relation to the length of stay.

	Length of Stay		
	0-30 days	>30 days	*p*-Value
Number of patients	3233	163	
LOS in days, Median (IQR)	2.63 (1-6.04)	46.00 (35.75-67.08)	<0.001
By Age Group, *N* (%)			<0.001
≤12 M	918 (28.39%)	73 (44.79%)	
>12 to ≤60 M	1,160 (35.88%)	45 (27.61%)	
>60 to ≤120 M	696 (21.53%)	28 (17.18%)	
>120 M	459 (14.20%)	17 (10.43%)	
Patient-related factors, Median (IQR)			
Height (CM)	87.0 (65.00–114.00)	73.00 (58.00–99.00)	<0.001
Weight (KG)	11.20 (6.30–18.70)	8.00 (4.60–13.70)	<0.001
Sex, *N* (%)			0.166
Male	1,766 (54.62%)	80 (49.08%)	
Female	1,467 (45.38%)	83 (50.92%)	
Patient Origin, *N* (%)			<0.001
Ward	1,021 (31.58%)	83 (50.92%)	
Emergency	1,113 (34.43%)	57 (34.97%)	
Another hospital ICU	87 (2.69%)	17 (10.43%)	
Operating room	973 (30.10%)	4 (2.45%)	
Other	39 (1.21%)	2 (1.23%)	
Previous PICU admission, *N* (%)	487 (15.06%)	130 (20.25%)	0.073

LOS, Length of Stay; ICU, Intensive Care Unit; PICU, Pediatric Intensive Care Unit.

Patient origin differed significantly between the two groups (*p* < 0.001). Patients admitted from the ward comprised the majority (50.92%) of LSPs, followed by patients admitted from the emergency room (34.97%; Table 1).

As estimated by PRISM III, LSPs had a significantly higher probability of death (1.08%%; *p* < 0.001). The actual mortality was as well significantly higher in LSPs (26.4%; *p* < 0.001), Most patients were admitted to the PICU because of respiratory diseases (29.77%), followed by infectious diseases (16.6%). Most LSPs were admitted with respiratory (51.53%) or infectious (22.09%) diseases (Table 2).

**Table 2:** Characteristics of admission according to the length of stay.

	Length of Stay		
	0-30 days	>30 days	*p*-Value
Number of patients	3233	163	
PRISM III probability of death (%)			
Median (IQR)	0.51 (0.29–1.48)	1.08 (0.56–3.25)	<0.001
Mortality, *N* (%)	179 (5.50%)	43 (26.40%)	<0.001
Admission diagnosis by category, *N* (%)			<0.001
CNS Disease	526 (16.27%)	27 (16.56%)	
CVD	54 (1.67%)	1 (0.61%)	
Respiratory Disease	927 (28.67%)	84 (51.53%)	
Infectious Disease	528 (16.33%)	36 (22.09%)	
Immunological Disease	22 (0.68%)	1 (0.61%)	
Metabolic/Genetic Disease	147 (4.55%)	3 (1.84%)	
Hematologic/Oncologic Disease	431 (13.33%)	3 (1.84%)	
Gastroenterological/Hepatic Disease	157 (4.86%)	2 (1.23%)	
Renal Disease	116 (3.59%)	3 (1.84%)	
Endocrine Disease	157 (4.86%)	1 (0.61%)	
Other Diseases	168 (5.20%)	2 (1.23%)	
Comorbidities present at admission, *N* (%)			
CNS Disease	1,044 (32.30%)	89 (54.60%)	<0.001
CVD	317 (9.80%)	26 (16.00%)	0.011
Respiratory Disease	743 (23.00%)	83 (50.90%)	<0.001
Infectious Disease	1,122 (34.70%)	111 (68.10%)	<0.001
Immunological Disease	25 (0.80%)	5 (3.10%)	0.002
Metabolic/Genetic Disease	504 (15.60%)	29 (17.80%)	0.451
Hematologic/Oncologic Disease	431 (13.30%)	16 (9.80%)	0.195
Gastroenterological/Hepatic Disease	412 (12.70%)	24 (14.70%)	0.461
Renal Disease	230 (7.10%)	19 (11.70%)	0.03
Endocrine Disease	218 (6.70%)	4 (2.50%)	0.031
Total Morbidities, Median (IQR)	1.00 (1.00–2.00)	2.00 (2.00–3.00)	<0.001

PRISM, pediatric risk of mortality score; CNS, central nervous system; CVD, cardiovascular disease.

Comorbidities at admission for LSPs were significantly more frequent than that for shorter stay patients, with median values of 2 (IQR, 2–3) and 1 (IQR, 1–2), respectively. LSPs had more comorbidities related to infectious diseases (68.10%), central nervous diseases (54.60%), and respiratory diseases (50.90%) compared to shorter stay patients with *p*-value < 0.001 (See Table 2 below).

### Events and support during admission

Procedure frequency, need for respiratory support, and complications during admission were predominant in LSPs. CVC, Foley catheter, nasogastric tube (NGT), PICC, arterial line, and ICP monitor/EVD insertions were significantly higher in patients who stayed for more than 30 days with 52.76%, 40.49%, 39.26%, 20.86%, 11.04% and 4.29%, respectively with *p*-value < 0.001. Furthermore, LSPs had more tracheostomies compared to other groups (27.61%) (*p* < 0.001; see Table 3 below).

**Table 3:** History of admission according to the length of stay.

	Length of Stay		
	0-30 days	>30 days	*p*-Value
Number of patients	3233	163	
Procedures during admission, *N* (%)			
CRRT	104 (3.22%)	7 (4.29%)	0.450
CVC insertion	460 (14.23%)	86 (52.76%)	<0.001
Exchange transfusion	27 (0.84%)	0 (0.00%)	0.241
Foley's catheter insertion	382 (11.82%)	66 (40.49%)	<0.001
ICP monitor/EVD	28 (0.87%)	7 (4.29%)	<0.001
PICC insertion	46 (1.42%)	34 (20.86%)	<0.001
Tracheostomy	71 (2.20%)	45 (27.61%)	<0.001
Arterial line insertion	78 (2.41%)	18 (11.04%)	<0.001
Respiratory support, *N* (%)			
High flow nasal cannula	513 (15.87%)	62 (38.04%)	<0.001
Non-invasive ventilation	464 (14.35%)	106 (65.03%)	<0.001
Conventional ventilation	923 (28.55%)	127 (77.91%)	<0.001
High-frequency ventilation	92 (2.80%)	30 (18.40%)	<0.001
Nitric Oxide	12 (0.40%)	2 (1.20%)	0.096
Complications during admission, *N* (%)			
VAP	8 (0.25%)	7 (4.29%)	<0.001
CLABSI	18 (0.56%)	27 (16.56%)	<0.001
CAUTI	6 (0.19%)	5 (3.07%)	<0.001
HAP	8 (0.25%)	7 (4.29%)	<0.001
Arrest During Admission, *N* (%)	116 (3.59%)	21 (12.88%)	<0.001
DNR status, *N* (%)	82 (2.50%)	28 (17.20%)	<0.001

CRRT, continuous renal replacement therapy; CVC, central venous catheter; ICP, intracranial pressure; EVD, extra-ventricular drain; PICC, peripherally inserted central venous catheter; VAP, ventilator-associated pneumonia; CLABSI, central line-associated bloodstream infection; CAUTI, catheter-associated urinary tract infection; HAP, hospital-acquired pneumonia; DNR, do not resuscitate.

LSPs also required more respiratory support during admission. High flow nasal cannula, non-invasive, conventional, and high-frequency ventilation were utilized in a significantly greater number of LSPs—38.04%, 65.03%, 77.91%, and 18.40%, respectively (*p* < 0.001; Table 3).

Regarding complications during PICU admission, out of the patients admitted for more than 30 days, 27 (16.56%) had CLABSI, while VAP, HAP, and CAUTI were reported for 4.29%, 4.29%, and 3.07%, respectively. Complications during PICU admission were significantly higher in patients with more than 30 days of hospital stay (*p* < 0.001; Table 3). During admission, LSPs were found to have significantly more arrest events (12.88%), and patient-signed DNR (17.20%; *p* < 0.001).

### Multivariate analysis

Multivariate logistic regression analysis was conducted for all variables that were significantly different between the groups. The remaining factors with significant correlations after the multivariate analysis are presented in Table 4. CAUTI, CLABSI, VAP, and procedures during admission, such as ICP monitoring, PICC insertion, and tracheostomy, had the highest OR compared to other variables included in the multivariate analysis.

**Table 4:** Multivariate analysis according to the length of stay Status.

	Odds Ratio	95% CI		
		Lower	Upper	*p*-Value
Patient origin				
Ward (Reference group)	Reference	Reference	Reference	<0.001
Emergency	1.11	0.70	1.76	0.657
Another hospital ICU	3.03	1.32	6.92	0.009
Operating room	0.12	0.03	0.44	0.001
Other	2.97	0.55	16.12	0.206
Comorbidities				
CNS Disease	1.68	1.07	2.63	0.024
Procedures during admissions				
ICP monitor/EVD	5.93	1.72	20.42	0.005
PICC insertion	5.67	2.64	12.20	<0.001
Tracheostomy	7.72	4.18	14.28	<0.001
Respiratory support during admission				
Non-invasive Mechanical Ventilation	4.92	3.12	7.75	<0.001
Conventional Mechanical Ventilation	1.83	1.09	3.06	0.022
High Frequency Oscillatory Ventilation	2.56	1.31	5.02	0.006
Complications during admission				
VAP	8.66	1.48	50.53	0.016
CLABSI	12.87	5.34	31.01	<0.001
HAP	4.88	1.12	21.29	0.035
CAUTI	9.16	1.62	51.97	0.012
DNR Status	5.55	2.95	10.44	<0.001

ICU, intensive care unit; CNS, central nervous system; ICP, intracranial pressure; EVD, extra-ventricular drain; PICC, peripherally inserted central venous catheter; VAP, ventilator-associated pneumonia; CLABSI, central line-associated bloodstream infection; CAUTI, catheter-associated urinary tract infection; HAP, hospital-acquired pneumonia; DNR, do not resuscitate.

Patients admitted as a referral from other hospitals' intensive care units had an OR of 3.03 (1.32–6.96, 95% CI; *p *= 0.009), while patients admitted postoperatively from the operating room had an OR of 0.12 (0.03–0.44, 95% CI; *p *= 0.001). Central nervous system (CNS) disease was the only comorbidity with significant relationships in the analysis, with an OR of 1.68 (1.07–2.63, 95% CI; *p *= 0.024; Table 4).

Procedures during PICU admission, including ICP monitor/EVD, PICC insertion, and tracheostomy, were found to significantly increase the LOS with ORs of 5.93 (1.72–20.42, 95% CI; *p* = 0.005), 5.67 (2.64–12.20, 95% CI; *p* < 0.001), and 7.72 (4.18–14.28, 95% CI; *p* < 0.001), respectively. Additionally, respiratory support required during admission also affected the LOS. Non-invasive ventilation had an OR of 4.92 (3.12–7.75, 95% CI; *p* < 0.001), conventional mechanical ventilation had an OR of 1.83 (1.09–3.06, 95% CI; *p* = 0.022), and high frequency oscillatory ventilation had an OR of 2.56 (1.31–5.02, 95% CI; *p* = 0.006; Table 4).

Complications during PICU admission had the highest impact on LOS. CLABSI had the highest OR among the variables included in the study (OR, 12.87; 95% CI 5.34–31.01; *p* < 0.001). While other complications such as CAUTI with an OR of 9.16 (1.62–51.97, 95% CI; *p* = 0.012), VAP with an OR of 8.66 (1.48–50.53, 95% CI; *p* = 0.016), and HAP with an OR of 4.88 (1.12–21.29, 95% CI; *p *= 0.035) also showed an association with increased LOS—although to a lesser degree than CLABSI. Patients with DNR status were also associated with a prolonged LOS with an OR of 5.55 (2.95–10.44, 95% CI); *p* < 0.001; see Table 4 below).

## Discussion

This study aimed to identify the factors influencing LOS in a tertiary PICU in Riyadh, Saudi Arabia. In this retrospective, single-center, observational study, the initial analysis demonstrated that patients with LSPs had significantly lower age and growth parameters. Additionally, admission source, diagnosis, comorbidities, severity scores, procedures, complications, and respiratory support required during admission were also significantly related to prolonged LOS. Moreover, multivariate analysis showed that admission source, CNS comorbidities, procedures, respiratory support, and complications during admission significantly influenced LOS.

Multiple studies have shown that patients requiring recurring PICU admissions have worse outcomes and complications (20). Infants (0–1 year old), those at risk of undernutrition, and those with multiple complex comorbidities are at a higher risk of morbidity and mortality. Children with low growth parameters and low body mass index (BMI) showed a higher rate of failed mechanical ventilation, leading to prolonged ICU stay (21). Although BMI and Z scores were not accounted for in this study, age, weight, and height were not significantly related to prolonged LOS.

Complex comorbidities corresponded significantly with LOS and mortality, specifically CNS and infectious diseases (1, 22–24). In a study by Obrien et al. CNS comorbidities were the most common in patients admitted to the PICU; however, they did not significantly increase LOS (24). In contrast, in this study, multivariate analysis revealed a significant relationship between CNS comorbidities and a prolonged LOS. This variation between the studies could be due to variations in population, intensive care facilities, and healthcare systems between the countries in which they were conducted.

Tripathi et al. found that children admitted from the ward had more complicated courses and longer LOS than those admitted directly to the PICU (25). The most common admission source associated with LOS differed between studies (13, 26). Some patients had a longer stay associated with those sourced from another facility, similar to our findings.

Admitted patients with respiratory diseases comprised the majority of the LSPs in this study. Comparably, multiple studies have revealed that respiratory disease is the most common cause of admission and mortality in PICUs (21, 24, 27). This could be due to the use of respiratory support and resulting potential complications (28), leading to an unexpected LOS. This was demonstrated by multivariate analysis in our study.

Additionally, the use of foreign devices and procedures corresponded to LOS, specifically the insertion of the CVC, Foley catheter, arterial line, and ICP monitor/EVD. These findings resonate with the trends observed in a study by Miura et al., wherein LSPs required similar foreign device insertions (13). We further observed that tracheostomy procedures were performed in patients with an increased LOS. A late tracheostomy decision was associated with a longer LOS and longer weaning duration. Characteristically, awaiting tracheostomy is associated with prolonged mechanical ventilation and complications (28).

Use of devices, such as mechanical ventilators, may increase the risk of HAIs and complications during PICU stay (7). Blood-stream infections are the most common in PICUs and account for 28%–52% of HAIs (29). CLABSI and VAP, as reported by a systematic review and other studies, are among the most common device-related infections in PICUs (6, 30). CLABSI had the highest impact on LOS in our study. Furthermore, CLABSIs are responsible for substantial mortality, morbidity, extended duration of hospital stay, and additional hospital costs (31). Multiple studies have demonstrated the effect of nosocomial infections on LOS (32), while only a few have demonstrated an independent relationship between VAP, CLABSI, and CAUTI or HAIs as a broad term to prolonged LOS in PICUs, as presented in our study (10, 33).

PRISM III, a severity scoring system, has an impact on LOS based on the disease severity. As the PRISM score increased, the median LOS also increased. Similarly, Pollack et al. demonstrated that the median LOS increased in parallel with mortality risk until a PRISM score of 20–25, where the median LOS decreased due to earlier mortality (1). The initial analysis in our study showed that LSPs had a significantly higher mortality risk, as predicted by PRISM III; however, this association was absent in the subsequent multivariate analysis. This can be attributed to the fact that PRISM III was originally developed using data from different populations and healthcare systems, raising the need for studies to validate its accuracy in our pediatric population.

DNR status was associated with a prolonged LOS, as demonstrated in a similar study (34). This may be attributed to the timing and rationale for withholding specific intensive support modalities and categorizing patients according to DNR status among different intensivists and institutions.

There are significant variations in DNR adoption based on region, religious beliefs, and the PICUs structure and advancement. More specifically, a lack of comprehension, religious beliefs, and culture leads to hesitancy and delay in withdrawing support within the region (35–37). Reluctancy in DNR orders and withdrawal of support may prolong the patient's clinical course. Furthermore, determining DNR for a critically ill patient with a high risk of death is guided by the expected quality of life and predicted comorbidities (38–40).

### Limitations

This study has several limitations. First, it was conducted at a single institution, limiting the generalizability of its findings. Moreover, this triggers questions of construct validity as well as concerns regarding the reliability and replicability of a study centered on a single institution. Second, the study was retrospective; thus, institutional practices and individualized physician care may have also influenced the study outcomes.

## Conclusions

Prolonged LOS demonstrated direct correlations with admission source, CNS disease comorbidity, and procedures performed during the PICU stay. In addition, respiratory support needed during PICU stay, CLABSI, VAP, CAUTI, HAP, and DNR status were all factors associated with prolonged LOS. These variables may serve as future prediction models for predicting the expected LOS. We recommend formulating a multicenter national study to aid the identification of similar factors.

## Data Availability

The raw data supporting the conclusions of this article will be made available by the authors, without undue reservation.
